# A Rare Case of Miller Fisher Syndrome in a 45-Year-Old Female

**DOI:** 10.7759/cureus.36387

**Published:** 2023-03-20

**Authors:** Ruthwik Duvuru, Shivani Raju, Faisal Nawaz

**Affiliations:** 1 Medicine, Mohammed Bin Rashid University of Medicine and Health Sciences, Dubai, ARE; 2 Neurology, The Oxford Medical College, Hospital & Research Centre, Bangalore, IND; 3 Psychiatry, Al Amal Psychiatric Hospital, Dubai, ARE

**Keywords:** demyelination syndrome, intravenous immunoglobulins (ivig), miller fisher syndrome (mfs), adult neurology, guillain- barré syndrome

## Abstract

Miller Fisher syndrome (MFS) is an uncommon form of Guillain-Barré syndrome (GBS), a neurological condition that is acquired, degenerative, demyelinating, and frequently characterized by gradual, symmetrical ascending paralysis. Ophthalmoplegia, ataxia, and areflexia are common symptoms that follow a bacterial or viral infection. Here, we want to draw attention to a rare case of MFS in a 45-year-old Indian female who had dysphagia, dysphasia, ataxia, and dyskinesia while moving around. Unusually, she had no past medical history of *Campylobacter jejuni *infection, recent vaccinations, upper respiratory tract infections, or any sexually transmitted diseases. Since this disorder has excellent prognosis, early diagnosis and effective treatment are crucial to minimizing unnecessary medical intervention and psychological suffering.

## Introduction

Miller Fisher originally characterized the condition of ophthalmoplegia, ataxia, and areflexia in 1956 [1]. Based on its areflexia, cerebrospinal fluid abnormalities, and post-infectious manifestation, this disease has long been thought to be a subtype of Guillain-Barré syndrome (GBS). Miller Fisher syndrome (MFS) differs from GBS in several key clinical features and presents an extensive and challenging differential diagnosis. It is critical to comprehend that MFS is both a variant of GBS and a separate condition with unique therapy requirements [2].

Although the usual clinical characteristics of GBS and MFS are well known, the contemporary classification schemes do not accurately capture the complete range of either condition. Based on the present understanding of the comparable pathophysiological characteristics of each illness manifestation, GBS and MFS are further classified into various subtypes: GBS is separated into incomplete (such as acute ophthalmoparesis and acute ataxic neuropathy), localized (such as pharyngeal-cervical-brachial weakness and bifacial weakness with paraesthesias), and CNS subtypes of MFS (Bickerstaff brainstem encephalitis) [3]. Clinical characteristics of these ailments are comparable, including a history of an earlier infection, a mono-phasic disease course, and symmetrical weakness in the head or limbs. Antiganglioside antibodies, cerebrospinal fluid albuminocytological dissociation (enhanced protein, normal cell count), and neurophysiological evidence of axonal or demyelinating neuropathy are contributing variables that, while frequently supportive of a diagnosis, should not be relied upon [4]. A special antibody that defines MFS is present in the majority of patients [5].

Paraparetic GBS, bifacial weakness with paraesthesias, acute ataxic neuropathy, acute ophthalmoparesis, acute ptosis, and acute mydriasis are uncommon indications of GBS-related diseases. In most cases of MFS, individuals have a positive prognosis [5]. Additionally, many neurologists might not be aware that up to 10% of GBS patients have deep tendon reflexes, some of which could even be brisk in expression, leading way to other complications [4]. For patient care and research such as clinical trials and studies on the safety of vaccines, accurate diagnostic criteria are crucial. There are several diagnostic standards for GBS that have been put forth, most recently by the Brighton Collaboration [6,7].

Physical therapy, plasma exchange, or plasmapheresis, a practice used to purify the blood, and intravenous immunoglobulin (IVIG) therapy, which employs purified plasma, are the most frequently utilized treatments for this illness. The recuperation starts two to four weeks after symptoms appear and can be practically fully complete in six months. However, some people continue to struggle with impairments. Rarely, relapses may happen in less than 3% of cases [5]. The management of pain is a pertinent factor and a deterrent to hospital recovery. Therefore, early in the course of the disease, an ideal pain regimen is crucial to speed healing. Due to the complex nature of the pain, it is usually essential to take a combination of pain relief drugs [8]. Physical therapy (PT)/occupational therapy (OT) are crucial components of MFS recovery and management. A suitable treatment plan can assist a patient in reducing discomfort, gaining strength and endurance, and avoiding further complications [9].

Here, we report a rare case of MFS in a middle-aged female and a subsequent treatment regimen that had an objective and subjective improvement in the patient's condition.

## Case presentation

A 45-year-old Indian female presented to the neurology outpatient clinic with a three-day history of numbness of all four limbs leading to difficulty in walking, difficulty in swallowing both solids and liquids, difficulty speaking, and swaying whilst walking. Past medical history is significant for hypertension, diabetes, tuberculosis, and coronary artery disease. She had no past medical history of *Campylobacter jejuni* infection, recent vaccinations, upper respiratory tract infections, or any sexually transmitted diseases. Upon physical exam, the patient was comfortable, conscious, oriented, and cooperative with normal vital signs. The cranial nerves exam was significant for bilateral horizontal gaze palsy, flaccid dysarthria, absent palatal movements, and difficulty swallowing. The rest of the examination was significant for dysmetria on finger-to-nose and alternating movements testing, but was less pronounced on heel-to-shin testing, absent Hoffmann’s, knee jerk, ankle reflexes, and decreased perception of touch, pain, and temperature below the knee. On gait examination, the patient also showed a steppage gait.

Subsequently, the patient was admitted to the neurology ward, with the following orders; laboratory: complete blood count, random blood glucose, renal function test, liver function test, and an electrolyte panel. She was given Intravenous (IV) normal saline 75 milliliters per hour, IV mannitol 100 milliliter, IV methylprednisolone 1 g in 100-milliliter normal saline over three hours, and per os (PO) ranitidine. Furthermore, she was referred for an ophthalmology review, a two-dimensional (2D) echo of the heart, audio gram, magnetic resonance imaging (MRI), and magnetic resonance angiogram (MRA) of the brain with contrast.

Her cardiac evaluation came out normal, however, her ophthalmology review gave an impression of bilateral sixth nerve palsy. Her initial laboratory orders resulted in normal values (Tables 1, 2, 3).

**Table 1:** Complete blood count on day 1 of admission

Test	Value
Hemoglobin	13.6 %
Red blood cell count	6.0 x 10^12^/L
White blood cell count	7.0 x 10^9^/L
Platelets	106,000/microliter

**Table 2:** Renal function tests and electrolytes on day 1 of admission

Test	Value
Urea	25 mg/dL
Creatinine	1.0 mg/dL
Sodium	130 mmol/L
Potassium	3.6 mmol/L

**Table 3:** Liver function tests on day 1 of admission

Test	Value
Total bilirubin	0.4 mg/dL
Direct bilirubin	0.2 mg/dL
Alkaline aminotransferase	26 U/L
Alanine aminotransferase	19 U/L
Alkaline phosphatase	58 U/L

MRI and MRA were reportedly normal and the audiogram revealed sensorineural hearing loss bilaterally. By day 2 of admission, we were looking at myasthenia gravis, peripheral demyelination disease, and MFS as our differentials, and therefore, PO neostigmine 50 mg was prescribed.

Later, a nerve conduction study (NCS) of all her four limbs, lumbar puncture (LP), and HIV testing was done on day 4 of admission. The patient was HIV negative, with normal nerve conduction study and increased protein in her cerebrospinal fluid as derived from LP.

Based on clinical findings of ophthalmoplegia, ataxia, areflexia, and findings of NCS along with LP, a diagnosis of MFS was established on day 5. Therefore the patient was started on IV immunoglobulin (IVIG) 0.4 gm/kg bodyweight for five days duration and plasmapheresis was performed on day 7 and day 9 of admission with the removal of 1200 mL of plasma at each session with subsequent administration of eight units of fresh frozen plasma.

Follow up on day 10, upon completion of her IVIG regimen, there was significant improvement in her ataxia, pupillary reactivity, patellar movement, swallowing, and lateral and down gaze palsy.

## Discussion

GBS is an umbrella term used for acute immune-mediated polyneuropathy conditions and is the most common cause of acute flaccid paralysis worldwide most often preceded by infection of the upper respiratory tract [10]. GBS has a male gender predominance with a region-dependent incidence rate ranging between 0.38 (95% CI 0.25-0.56) to 2.53 [95% CI 1.87-3.56] per 100,000 and peaks during arboviral disease outbreaks. GBS occurs at a mean age of 43.6 years and the incidence risk increases with age [11,12].

Diagnosis is usually based on clinical history, and over 60% of patients described antecedent infectious symptoms [13]. However, our patient didn’t have any preceding symptoms of illness and wasn’t even vaccinated for the coronavirus disease 2019 (COVID-19) virus or seasonal flu. Although Initial symptoms include progressive symmetrical paraesthesias, patients can also complain of distal numbness and back pain similar to our case. Other symptoms include distal weakness, and absent deep tendon reflexes [13].

Amongst the subtypes of GBS, MFS presents with progressive bilateral ophthalmoplegia, ataxia, and areflexia as the characteristic clinical presentation [14]. The most common differentials for MFS include myasthenia gravis and brain stroke; however, both of them could be excluded if fatiguability and acute onset of symptoms are present [11].

MFS symptoms improve over a while without any intervention; however, IVIG and plasmapheresis are most commonly used. Corticosteroids such as IV methylprednisolone can sometimes be used as an adjunct to IVIG. The recommended IVIG dosing is generally 2 g/kg body weight given over five days, and the same dosing was maintained in our patient at 0.4 g/kg body weight [15].

Following treatment, the prognosis is dependent on several factors, including but not limited to the age of the patient, disease progression rate, need for ventilation, and proof of axonal degeneration on electrophysiological studies [16]. Mori et al. reported that irrespective of these factors, ataxia, and ophthalmoplegia disappeared in a median period of one to three months after symptom onset and all the patients in their study had little to no disability at six months mark [17].

## Conclusions

MFS, a variant of GBS, can also occur in patients with no history of *C. jejuni* infection, recent vaccinations, upper respiratory tract infections, or any sexually transmitted disease. Diagnosis and subsequent treatment of this condition are very important as they can reduce unnecessary treatment and psychological distress as the disease has a very good prognosis.
